# Supplementary material for: A time of decline: An eco-anthropological and ethnohistorical investigation of mpox in the Central African Republic
Source: PLOS Glob Public Health. 2024 Mar 22;4(3):e0002937. doi: 10.1371/journal.pgph.0002937 (PMC10959331; doi:10.1371/journal.pgph.0002937)
Supplement: Attachment — Submitted filename: Peer review comments from Mateus.docx [file pgph.0002937.s002.docx]

**Peer review comments from M**

**Title:**

It is important to have the full word Mankeypox instead of mpox => **A time of decline: an eco-anthropological and ethnohistorical investigation of Mankey-pox (mpox) in the Central African Republic.**

Line 6-11: It is good to number affiliation institutions from 1 to 4, so that is clear to understand the affiliation of each author.

**Abstract:**

Line 25: Is it really a mixed method or just qualitative study using different approaches. If this is the case, then delete the word mixed.

Line 29: … ethnohistorical, anthropological interviews in Sango and French and conducted participant-observation… If Sango is a language in CAR, then good to say “interviews in two languages: Sango and French. This helps even people who have been to CAR to understand the sentence.

**Introduction:**

Line 47: Need for a reference after endemic

Line 48: Need a reference after (clade II).

Line 64: The word “hereafter” is not necessary.

Line 68: Is the word “some” important in front of 95. Does it mean this number is not certain?

Line 85-87: The sentence “As part of the multidisciplinary AFRIPOX project, we conducted an eco-anthropological, 86 ethnohistorical investigation in two regions of the CAR with different frequencies and case numbers of 87 mpox outbreaks” should be part of methodology.

It is important to add information about risk factors of transmission between humans, humans-animals and local behavours. The authors say the disease is endemic in CAR, that means people have been exposed to it for long time. So, what is being done to respond to this public health issue at the community level and at the national level (health system)?

**Materials and methods**

Line 93: is it really a mixed method or just qualitative study.

Line 96 – 112: starting from the sentence: The territory now delineated … this part should be in the introduction.

From Line 132 in the whole method section: is it important to specify author 1, author 5, author 6,…? It is better to use general language as team to avoid frustration among authors.

Line 141: What does this sentence mean: …occasionally, children or other adults would listen to the exchanges? Were they part of the study participants or not? If no, why should they listen to the conversation? If yes, were children targeted for this study? And did it get the necessary approval for vulnerable group?

Line 142: … participants authorization… => I hope this means participant consent. Use the commonly known language. Was the consent verbal or written? Did they sign consent form? Please specify if participation was voluntary or paid.

Line 143: We did not seek saturation but sought to maximize the number of interviews for both sites. => How many participants per sites and how the participants were selected (sampling method)?

Line 150-152: Please specify here what you mean by authorization and oral consent. Use the commonly known language for ethics.

Line 155-159: The word survey just appear suddenly in the middle of the text. This means the study was really mixed methods. However, it is not clear at the beginning. This should be stated everywhere it is said mixed method and in the abstract. How did the authors select participants for the survey (sampling method)? And why 24 participants? Is this number sufficient for a survey? Was there a sample size calculation? How?

Line 157: ” Because of very high mobility among village residents at the time,…” what time?

Line 157-159: “Because of very high mobility among village residents at the time, participants were contacted in person periodically over the study period to provide dietary, activity and income data; data was therefore not collected for all 24 participants for the entirety of the six-month data collection period.” Please resolve issue of grammar in this sentence and you can slip it into 2.

Line 160: It is better to start this paragraph by explaining that you used thematic analysis then continue explaining how as written here. Was there any conceptual framework used?

Lines 160-169: It is not clear how the thematic analysis was conducted. Inductive or deductive method? It is also not clear which data were anaylised with Nvivo (qualitative or survey) and which ones were analysed using R software (qualitative or survey)?

Ethics

Line 171-178: If I understand well, written consent was not suggested because of safety to avoid mpox transmission. If that is the case, then it should be clearly stated here, so the readers do not have to assume. This section does not say anything about consent for survey questionnaire. Was it not needed? Why?

Information about ethics were stated many time in the previous section in the method. It is important to separate them and put everything about ethics in this section (171-178).

**Results**

Line 180-186: This paragraph should be part of the methods

Line 212: Is this “Republic of Congo or Democratic Republic of Congo”? They are two different countries. Be sure which you refer to here.

Line 218-221: Is this a quote? If yes, it should have quotation mark “….”

Line 234: What do authors mean by “meat hunger”?

Line 235-237: This should be in a quote format.

Line 275-281: Are these data reported here part of this study? If yes, it should be stated clearly from the beginning and in the methods. If no, is there an unpublished report that the author refer to? It could be better to have this information in the introduction as part of the literature to support the rationale.

Line 295: Add quote mark at the beginning.

Line 308-315: Put quote mark.

Line 332-339: Put quote mark.

Line 349-354: Put quote mark.

Line 356-358: If this is a quote, then show it.

Line 375: …Lots of illnesses… should read Lot of illnesses…

Line 378-381: Please put quote marks here. And all the quotes should have the same format.

Line 389-392: Please put quote mark.

**Discussion**

Line 400-408: This paragraph has a mix of many ideas. It would better to separate paragraphs by ideas (themes).

Line 430-431: Difficult to understand why sleeping sickness is referenced here.

Line 434-442: Can the message here be made clear? It can be interpreted that WWF made a mistake in its awareness campaign.

Line 490: Is the word “see” relevant here?

Line 498: The sentence “…further multi-disciplinary investigation…” should read “…further multi-disciplinary investigations…”

Line 528-529: “This unpublished data was collected for a different study. It offers, however, an indicator of changes in meat consumption over the past 30 years.” => fix issue of grammar in this sentence.

**Conclusion**

Line 545: Good idea of co-development of solutions with local populations. However, this come only in the conclusion. Nothing is seen in the results and rationale about it.

Line 547: Why reference in the conclusion?

Line 549-554: Acknowledgement should at the very beginning before abstract.

Line 556: Is this Bibliography or just a list of references?

**General comments:**

- Fix the issue of page number formatting
- All the titles are too small and do not comply with PLOS GPH title guideline.
- The manuscript is scientifically relevant and carry the message that can inform policy and programs to improve public health interventions. Following the above review, the corrections are important to be made before further considerations.
