# Supplementary material for: A time of decline: An eco-anthropological and ethnohistorical investigation of mpox in the Central African Republic
Source: PLOS Glob Public Health. 2024 Mar 22;4(3):e0002937. doi: 10.1371/journal.pgph.0002937 (PMC10959331; doi:10.1371/journal.pgph.0002937)
Supplement: Attachment — Submitted filename: PLoS GPH ethno mpox.docx [file pgph.0002937.s003.docx]

PLoS GPH

Thank you for the opportunity to review the paper. It is a very interesting read, and gave different perspective to the mpox emergence in one of the most challenging context, the Central African Republic. It is of note that the paper dislodge the biomedical superiority as the only lens used to understand and control a ‘disease’, acknowledging the people’s story and narrative of what afflicted them. Hence I commend the team to spend time in this important piece of evidence and further increase awareness of much needed multidisciplinary approach to a complex problem.

The study is an investigation conducted in 2 regions in CAR with different endemicity and outbreak proneness related to mpox. Data was collected through interviews, participant observations and comparison with data from 30 years ago.

Certainly, it is a long read and I wonder how much has been cut as well as not to bore the reader but keep them engaged. This is a type of study that requires the reader to step out of the strict qualitative research methodologies and appreciate the richness of information.

My suggestions to the research team to improve the manuscript are as follows:

- Line 25: the term mixed method -does this refer to quantitative and qualitative assessments or in just to say different methods were deployed?
- Line 30-31: a database of historical data is mentioned, but with one time point only i.e 30 years ago; is there no data from the time in between 1993 and 2022?
- Line 33-34: The sentence ‘We also find important differences..’ belongs closer to Line 38 (.. reveals variability..) and not the similarity of the two sites in terms of their declension narrative
- In the introduction, line 66 onwards – what is the ‘outbreak’ term refers here i.e what is the threshold of declaring it as outbreak in CAR? Is it a notifiable disease that must be reported to the health authority? Is one case enough to declare so?
- Reference given in that paragraph is duplicate: reference 3 and 19 is the same
- Line 102: The CAR – suggest removing the word ‘The’
- Line 117-119: these 3 last sentences is unclear to read, suggest rephrasing
- On material and methods:
- Given the high mobility of the population, and the diversity of the tribes, how did selection bias be mitigated?
- What was the reason not to seek saturation during the data collection especially with sampling strategy of opportunistic participant recruitments and snowballing (asking key informants)
- How do you differentiate the reporting of individual versus group interviews or both are combined in Table 1? Why not doing focus group discussion?
- Participant observation time seems quite short, would there be any reasons for this?
- I appreciate clarity on the positionality and reflexivity of the research team
- In results, the quotes are organized sometimes integrated in paragraph, sometimes not, please standardized (example: line 235,, 258, 345..)
- Line 261 – is supplemental file 1 a text documenting rodent trapping techniques? (supplemental file I see is the video if not wrong)
- Line 274 beyond – the description of eating meat habits may be more specific? Meat days means once per day? Per person or per family? Is it the size change of the animals hunted in 1993 the main thing we need to understand?
- Line 301 and elsewhere: is the word ‘moral’ used as ethical understanding that relates to religion or beliefs in this population?
- The discussion is well written but the implications can be theorized – e.g would paragraph starting line 419, would mean the messages at least to seek care should consider the nosological terms? Or would health workers be more aware and alert on the description of symptoms and also for notification of cases?
- Please clarify if the Ebola messaging have influenced the way mpox is perceived or experienced being not lethal disease?
- The discussion is well written, albeit could benefit

It is an interesting read and definitely help us appreciate the complexity of such disease ‘emergence’. Not the easiest read as well so I hope the feedback above can improve the manuscript’s readability.
