# Supplementary material for: A time of decline: An eco-anthropological and ethnohistorical investigation of mpox in the Central African Republic
Source: PLOS Glob Public Health. 2024 Mar 22;4(3):e0002937. doi: 10.1371/journal.pgph.0002937 (PMC10959331; doi:10.1371/journal.pgph.0002937)
Supplement: Attachment — Submitted filename: Response to reviewers.docx [file pgph.0002937.s004.docx]

**Comments to the Author**

Please note that all references to Line numbers correspond to the CLEAN COPY of the revised manuscript.

**REVIEWER #1:**

**Title:**

It is important to have the full word Mankeypox instead of mpox => A time of decline: an eco-anthropological and ethnohistorical investigation of Mankey-pox (mpox) in the Central African Republic.

*In 2022, the World Health Organization designated “mpox” as its official term for this disease and virus. It encourages the use of “mpox” over “monkeypox”, because “mpox” avoids stigmatization or other potentially negative consequences associated with references to monkeys. For these reasons, we employ the term “mpox”. We have added “monkeypox” in the keywords to facilitate searches.*

*To our knowledge, “mankeypox” (employed twice by Reviewer 1) is not a commonly employed term in biomedical or public health literatures.*

Line 6-11: It is good to number affiliation institutions from 1 to 4, so that is clear to understand the affiliation of each author.

*Corrected*

**Abstract:**

Line 25: Is it really a mixed method or just qualitative study using different approaches. If this is the case, then delete the word mixed.

*This paper is primarily an ethnographic, ethnohistorical paper, but it does use limited quantitative data and analysis. We make this point clear in the Methods section. But we have deleted the “mixed methods” to avoid confusing readers.*

Line 29: … ethnohistorical, anthropological interviews in Sango and French and conducted participant-observation… If Sango is a language in CAR, then good to say “interviews in two languages: Sango and French. This helps even people who have been to CAR to understand the sentence.

*We modified the text to indicate Sango and French in the abstract.*

**Introduction:**

Line 47: Need for a reference after endemic

*The term “endemic” is used in highly varied ways. For certain publications, mpox is endemic in central and west Africa, whereas for others, it first emerged in Democratic Republic of Congo and became endemic there. Rather than to immerse ourselves from line 1 in debates about the meanings of endemicity, we have altered the sentence to reflect that there are frequent outbreaks but included a citation to support that claim.*

Line 48: Need a reference after (clade II).

*Citation added.*

Line 64: The word “hereafter” is not necessary.

*Corrected*

Line 68: Is the word “some” important in front of 95. Does it mean this number is not certain?

*Technically, in American English, the use of “some 95” simply means that there were 95 cases. The usage is correct, but we have deleted the term “some” so as not to confuse readers.*

Line 85-87: The sentence “As part of the multidisciplinary AFRIPOX project, we conducted an eco-anthropological, ethnohistorical investigation in two regions of the CAR with different frequencies and case numbers of mpox outbreaks” should be part of methodology.

*It is standard practice in an introduction to present a problem/question and to summarize in one line the type of study that was conducted that can offer an answer to that problem. We therefore moved the phrase about the multidisciplinary project to the Methods and Materials section. But we have kept the remaining content of this sentence in the introduction because it follows the convention of introducing the study in an introductory section.*

It is important to add information about risk factors of transmission between humans, humans-animals and local behavours. The authors say the disease is endemic in CAR, that means people have been exposed to it for long time. So, what is being done to respond to this public health issue at the community level and at the national level (health system)?

A. *We cannot respond to this question about risk factors because a full epidemiological analysis in Central African Republic has never been done to identify risk factors. Three co-authors (Authors 3, 4, and 6) on this article -- leaders in studies on mpox in CAR -- have insisted that these risk factors have never been identified. To ensure that readers understand this point, we have indicated this absence of identified risk factors. That said, we also list in Lines 70-73 the characteristics that appear to be associated with transmission.*
B. *On the question of endemicity, it would be important to note that endemicity is not necessarily about long-term exposure. Although the meaning of this term is debated, it does mean that the* ***presence of the virus is relatively constant****. Because of potential confusion, we have deleted the term from our paper.*
C. *Finally, we would respectfully contend that adding national and community response to the Introduction would distract readers from the central purpose of the Introduction. We have therefore added additional text on national response in lines 126-131, followed by some local-level features of mpox response.*

**Materials and methods**

Line 93: is it really a mixed method or just qualitative study.

*Again, it is mixed method, but because the study is mostly qualitative, we have deleted mixed to avoid confusion*.

Line 96 – 112: starting from the sentence: The territory now delineated … this part should be in the introduction.

*We respectfully disagree. This is contextual information that rightfully belongs in Materials and Methods. A Materials and Methods section should explain something about the site and context of the study. As an ethnohistorical study, we would contend that this historical summary must be included in this section so that our ethnohistorical results and analyses make sense to readers. Moreover, this inclusion here permits us to respond more effectively to the suggestions of Reviewer 5.*

From Line 132 in the whole method section: is it important to specify author 1, author 5, author 6,…? It is better to use general language as team to avoid frustration among authors.

*We respectfully disagree. Different co-authors made different contributions to the manuscript. In the interest of transparency, and in line with PLoS Global Public Health policy and COREQ reporting guidelines for qualitative research reporting, we have made clear throughout the manuscript the roles played by each author.*

Line 141: What does this sentence mean: …occasionally, children or other adults would listen to the exchanges? Were they part of the study participants or not? If no, why should they listen to the conversation? If yes, were children targeted for this study? And did it get the necessary approval for vulnerable group?

*Children were not part of the study and were thus not included as participants in interviews. Nevertheless, in our multiple decades of experience of conducting anthropological interviews in rural areas of central Africa, we have found it impossible to regulate the comings and goings of people, including children, who want to listen to an interview. Members of the research team sought to respect the time and other obligations of our participants, who sometimes relaxed, cooked food, cared for children, or did other chores while participating in our interviews. Insisting that children or other adults leave during our interviews would have expressed gross disrespect for our informants and their families. Moreover, the act of listening to family members or friends participate in an interview does not constitute participation in a study. It therefore does not require informed consent on the part of the participant.*

Line 142: … participants authorization… => I hope this means participant consent. Use the commonly known language. Was the consent verbal or written? Did they sign consent form? Please specify if participation was voluntary or paid.

*We replaced the term “authorization” with that of “consent”. Yes, we do mean consent. This consent was verbal, and participation was always voluntary, not paid.*

Line 143: We did not seek saturation but sought to maximize the number of interviews for both sites. => How many participants per sites and how the participants were selected (sampling method)?

*We have added information about recruitment procedures on Lines 167-172 to supplement the description of recruitment procedures.*

*Nevertheless, the number of interviews in qualitative investigations is conventionally included in the Results, not the Methods. We have kept this information in the Results section. In line with the Reviewer’s recommendation, we have indicated the number of interviews in both sites.*

Line 150-152: Please specify here what you mean by authorization and oral consent. Use the commonly known language for ethics.

*We regret the confusion that this line may have caused. In line with Reviewer 1’s recommendation, we have modified the sentences to clarify the fact that the researcher obtained consent for video recording. We also moved all information pertaining to ethics to the section on Ethics (lines 200-211).*

*We would respectfully submit that the terms used in reference to ethics are conventionally used and accepted ones in research ethics. There may be some regional differences, which may have either led to our use of certain terms or this Reviewer’s questions. We have thus modified our language to minimize any confusion.*

Line 155-159: The word survey just appears suddenly in the middle of the text. This means the study was really mixed methods. However, it is not clear at the beginning. This should be stated everywhere it is said mixed method and in the abstract. How did the authors select participants for the survey (sampling method)? And why 24 participants? Is this number sufficient for a survey? Was there a sample size calculation? How?

*We have deleted the term “mixed methods” because it appeared to cause this Reviewer considerable confusion. Again, the study is primarily ethnographic and ethnohistorical but there are quantitative data and analyses. We have deleted all references to mixed methods.*

*We have added additional information on convenience sampling, on reasons for recruiting participants. We included 24 participants because this was the number of people in the village willing and available to participate. We did no sample size collection because the surveys were part of a broader qualitative investigation.*

*We would argue strongly for the inclusion of this survey data. It provides an indicator of changes in wild meat hunting and consumption practices in the past 30 years. Without this data, we have no evidence for such changes other than what contemporary participants claim. We have spent many years working on oral historical evidence and remembering, recognizing that recollections are crucial, rich, and flawed evidence. The survey provides concrete data about these changes.*

Line 157: “Because of very high mobility among village residents at the time,…” what time?

*We specified the timing.*

Line 157-159: “Because of very high mobility among village residents at the time, participants were contacted in person periodically over the study period to provide dietary, activity and income data; data was therefore not collected for all 24 participants for the entirety of the six-month data collection period.” Please resolve issue of grammar in this sentence and you can slip it into 2.

*According to our knowledge of American English (corresponding author’s native language), there is no grammatical problem with this sentence. Nevertheless, we have modified the sentence, breaking it into two sentences so that it does not raise questions for readers.*

Line 160: It is better to start this paragraph by explaining that you used thematic analysis then continue explaining how as written here. Was there any conceptual framework used?

*We modified the paragraph to begin with the point about thematic analysis. We did not use an additional conceptual framework underpinning the thematic analysis, but this is not necessary to make the arguments we do in this manuscript.*

*More broadly, we highlight in the Introduction social sciences critiques of One Health and the importance of “nonscientific knowledge” in shedding light on local understandings and practices for explaining mpox epidemics.*

Lines 160-169: It is not clear how the thematic analysis was conducted. Inductive or deductive method? It is also not clear which data were analyzed with Nvivo (qualitative or survey) and which ones were analyzed using R software (qualitative or survey)?

*We apologize for any confusion and have modified the description of our thematic analysis to respond to these questions. In Lines 192-193, we specified that our qualitative evidence was entered into NVivo, that our coding was inductive. We also clarified in Lines 197-198 that our quantitative data concerning meat consumption from 1993 was entered into an excel file and that we performed descriptive statistics using R software.*

**Ethics**

Line 171-178: If I understand well, written consent was not suggested because of safety to avoid mpox transmission. If that is the case, then it should be clearly stated here, so the readers do not have to assume. This section does not say anything about consent for survey questionnaire. Was it not needed? Why? Information about ethics were stated many times in the previous section in the method. It is important to separate them and put everything about ethics in this section (171-178).

*We state explicitly the reasons for oral consent on Lines 204-205: “We obtained oral consent because of the social sensitivity of mpox and of anthropological research in CAR.” It appears that Reviewer 1 misunderstood the point here. The oral consent was not to avoid mpox transmission. We have modified the sentence to clarify.*

*As this Reviewer recommended, we have consolidated all mention of ethics in this section.*

*Finally, ethical approval for the survey was not required at the time, either by the Central African Republic, which had no ethics committee at the time, nor by Author 6’s university because the research was part of an ethnohistorical study. We have noted this point on Line 210-211. (The author did receive official authorization to conduct this research from what was then the Ministry of Research in the Central African Republic, although we no longer have a copy of that document. It would be important to note that ethical requirements have changed considerably in 30 years).*

**Results**

Line 180-186: This paragraph should be part of the methods

*In our experience, interviews numbers are an accepted part of the Results. We did not set out to interview 40 people. We have therefore kept this paragraph as is.*

Line 212: Is this “Republic of Congo or Democratic Republic of Congo”? They are two different countries. Be sure which you refer to here.

*We are certain that this reference is correct. It is the Republic of Congo, not DRC.*

Line 218-221: Is this a quote? If yes, it should have quotation mark “….”

*We have made changes here and throughout the manuscript to convene to PLOS GPH formatting conventions. All quotations are thus italicized and in quotation marks.*

Line 234: What do authors mean by “meat hunger”?

*To avoid confusion, we have included in this sentence a definition of “meat hunger” as “a lack of wild meat”*

Line 235-237: This should be in a quote format.

*Corrected.*

Line 275-281: Are these data reported here part of this study? If yes, it should be stated clearly from the beginning and in the methods. If no, is there an unpublished report that the author refer to? It could be better to have this information in the introduction as part of the literature to support the rationale.

*We apologize for any confusion here. We have explained clearly in the Methods section* *precisely what was collected and when it was collected. There is no unpublished report from this work.* *We also reformulated the sentences in Lines 322-324 to make clear that this glimpse into past meat consumption practices is based on data collected in 1993.*

Line 295: Add quote mark at the beginning.

*Corrected.*

Line 308-315: Put quote mark.

*Corrected*

Line 332-339: Put quote mark.

*Corrected*

Line 349-354: Put quote mark.

*Corrected*

Line 356-358: If this is a quote, then show it.

*This was an expression repeated by multiple informants.*

Line 375: …Lots of illnesses… should read Lot of illnesses…

*We have not corrected this because it is grammatically incorrect to say “Lot of illnesses…” Had we written “A lot of illnesses”, we would concur with this correction.*

Line 378-381: Please put quote marks here. And all the quotes should have the same format.

*Corrected.*

Line 389-392: Please put quote mark.

*Corrected.*

**Discussion**

Line 400-408: This paragraph has a mix of many ideas. It would better to separate paragraphs by ideas (themes).

*We respectfully disagree with Reviewer 1. In keeping with conventional structures of articles, this first paragraph provides a general summary of the main results that will be addressed in the Discussion. We address each of these themes in succession throughout the rest of the Discussion. We have also added subheadings within the Discussion section to facilitate better comprehension.*

Line 430-431: Difficult to understand why sleeping sickness is referenced here.

*We regret that this point was not as clearly framed as it could have been. This final sentence in this paragraph was simply an illustration of how lay publics creatively expand terms refer to wide-ranging illnesses for which biomedical researchers and clinicians have distinct terms and etiologies. We have deleted the sentence to avoid reader confusion and have integrated the reference with the two others that are cited.*

Line 434-442: Can the message here be made clear? It can be interpreted that WWF made a mistake in its awareness campaign.

*We are cannot judge whether the WWF made an error or not, and indeed, such a judgment would be beyond the scope of this manuscript. Rather, we highlighted that this local interpretation was an “unintended consequence” of the awareness campaigns. We have added additional language to make this point clearer (Lines 512-515).*

Line 490: Is the word “see” relevant here?

*This word is unnecessary and has been deleted.*

Line 498: The sentence “…further multi-disciplinary investigation…” should read “…further multi-disciplinary investigations…”

*This was not an error. It is grammatically acceptable to in English to write “further multi-disciplinary investigation”.*

Line 528-529: “This unpublished data was collected for a different study. It offers, however, an indicator of changes in meat consumption over the past 30 years.” => fix issue of grammar in this sentence.

*We have altered the sentence for clarity and readability.*

**Conclusion**

Line 545: Good idea of co-development of solutions with local populations. However, this come only in the conclusion. Nothing is seen in the results and rationale about it.

*We mention this co-development of solutions as a way of moving forward with the research that we conducted. Nevertheless, as desirable as this initiative would be, this type of co-development was not within the scope of our ethnohistorical study, nor of the overall study of which our work was a part. For that reason, it neither appears in the Methods, Results, or justification of the study.*

Line 547: Why reference in the conclusion?

*We deleted the reference.*

Line 549-554: Acknowledgement should at the very beginning before abstract.

*PLOS GPH guidelines specify that Acknowledgements should be included after the manuscript text and before the References.*

Line 556: Is this Bibliography or just a list of references?

*Our apologies for any confusion. We have altered this title to References.*

**General comments:**

- Fix the issue of page number formatting

*We understand that some reviewers, including this one, may have viewed problems with page numbers. Our version, however, doesn’t seem to present a problem with page number formatting, so it is possible that this error occurred in the process of uploading the manuscript to the PLOS GPH site. We apologize sincerely for this inconvenience to all reviewers and will try to the best of our ability to ensure that this problem does not recur with the resubmission.*

- All the titles are too small and do not comply with PLOS GPH title guideline.

*We have corrected the font size to comply with journal guidelines.*

- The manuscript is scientifically relevant and carry the message that can inform policy and programs to improve public health interventions. Following the above review, the corrections are important to be made before further considerations.

*We thank this Reviewer for an attentive reading and critical comments, which we think have made for a clearer and stronger manuscript.*

**REVIEWER #2**:

This mixed methods study used ethnohistorical and eco-anthropological analyses based on ethnographic work in two CAR regions to understand the political economies of mpox, and underscores the continued importance of anthropological work in the space of outbreaks and outbreak response. It is an excellent and important piece of work which could be further strengthened with some substantive revisions.

Firstly - I think it’s very difficult to speak about political economy without mentioning colonial history, particularly in a former French colony (and continued French intervention). What role might this history play in the wider political economy of mpox?

*This point is an excellent one, and we agree entirely. To that end, we have included a fuller description of the French colonial history and continued French intervention in the Methods and Materials section. In our Discussion, we have also expanded the historical dimensions of intensive wild meat hunting, consumption, and marketing (Lines 532-534) as well as multiple historical underpinnings of local emphases on decline as a catalyst for mpox emergence (Lines 592-599).*

Consider spending a little more time discussing dynamics related to human-to-human transmission, as mentioned in the introduction. Thinking of other mpox-endemic areas, like Nigeria, has there been similar spread from rural areas to urban? Perhaps something to signpost, not necessarily go in-depth here, but do any of your findings speak to this?

*We agree with Reviewer 2 that this dimension has been relatively neglected in our manuscript. We have added additional information about the primarily rural nature of mpox epidemics in CAR in the Introduction (Lines 76-77). We have also mentioned human-to-human transmission elsewhere, in Line 239.*

Some minor comments:

- This is a very well-written article, but please check some of the English language words as the meaning may be lost or misinterpreted, for example:

*We apologize for the errors that slipped through and thank the reader for this careful reading.*

o e.g., page 4 – line 55, ‘manipulation’ – does this mean butchering?)

*Manipulation means more than butchering. It can involve handling for preparation and marketing of meat. We therefore altered the language here, deleting manipulation to ‘handling and butchering”*

o page 4, line 61 – instead of ‘weakened,’ consider ‘insufficient’

*We have changed “weakened” to “insufficient” and have made additional changes in that sentence.*

- Page 5, line 69 – any deaths noted?

*Yes. We have added 12 deaths to this line (line 69).*

- Page 6 – can you add details about the colonial history and how this likely laid the groundwork for the dynamics (conflict, etc.) that you describe? I am sure that French colonialism played a big role in these current dynamics, please add detail here, otherwise it reads quite negative of the CAR.

*We entirely agree with Reviewer 2. We had earlier included this information, but in the interests of keeping down the word count, we deleted it prior to submission. But given these questions, we have reintegrated this historical summary of colonial rule.*

Page 8, Line 142 – participant ‘consent’ instead of authorization?

*Yes, we have altered the language to indicate consent.*

- Page 8, line 146 – were individuals suspected or lab confirmed mpox cases?

*Yes, it was laboratory confirmed. We have added the citation for this report.*

- Page 8, line 163 – do you mean ‘thematic’ codes, rather than large codes?

*Yes, with our apologies for the lack of clarity. We had wanted to distinguish between mother codes and subcodes. That said, we decided that additional information was not necessary and have simply referred to thematic codes.*

- Page 8, Line 165 – do you mean skin diseases, not cutaneous?

*We use these terms as synonyms, and to our knowledge, skin disease and cutaneous disease are terms that have the same meaning.*

- Line 213 – what is NTFPs? Please spell out all acronyms the first time it’s mentioned.

*We included an explanation of Non-Timber Forest Products when this term was first mention (Line 146-147).*

Line 234 – what is meat hunger?

*We explained this term in the same sentence as “lack of wild meat” (line 272).*

In terms of the section on livelihoods, hunting, etc. – you may want to add more to the first paragraph about why this section relates to mpox transmission (possibly).

*We agree that this point could be better spelled out. We therefore added a couple of lines to explain why examining livelihoods and hunting would be important to understand possible routes of exposure to animals carrying the virus (Lines 267-269).*

The section on local historical narratives and ‘blockages’ is very interesting, but perhaps we need more background about cultural context. In what situations might occultism be used?

*We have added additional explanation about occult forces as an explanation of misfortune, notably when affecting people previously in good health, or affecting several family members simultaneously Lines 377-382)*

Also, I would like to see more explicit attention to power and power inequalities within your political-economic analysis. What or who do people blame for the general state of decline? You mentioned the politicians, but what are these ethnic groups’ experiences of mainstream politics, are they a politically or socially marginalized group?

*We agree with Reviewer 2 that we needed to address this point. We have therefore added additional text in this section. Lines 407-411 provide a fuller explanation of the quotation mentioning earlier leaders David Dacko and Jean-Bedel Bokassa, and the subsequent marginalization of populations living in the Lobaye prefecture (and specifically Ngbaka peoples). In Lines 419-426, we provide additional explanation of the second quote, situating the quotation in terms of elite access to power and further accumulation of wealth at the expense of Lobaye, “southern” and non-elite populations. We also draw out further implications of these insights in the Discussion starting on Line 474.*

One other thing to consider, perhaps in the discussion. How do your findings problematize or shed light on the global health security agenda, and how/when certain diseases are declared a ‘public health emergency’? How do these heterogeneous experiences, particularly in endemic regions (vs the global multicountry outbreak), shape when and how something is declared an emergency? And then how does this shape response, funding flows, etc.? Some reflections on these points could add strength to the global importance of the work.

*This is a compelling and powerful observation, for which we sincerely thank Reviewer 2. We have added a full paragraph reflecting on the global health security agenda and its neglect of this recurrent health problem in low-income countries until 2022, when more widespread transmission affected high income countries. These reflections appear in Lines 600-610.*

**REVIEWER #3:**

Thank you for the opportunity to review the paper. It is a very interesting read, and gave different perspective to the mpox emergence in one of the most challenging context, the Central African Republic. It is of note that the paper dislodge the biomedical superiority as the only lens used to understand and control a ‘disease’, acknowledging the people’s story and narrative of what afflicted them. Hence, I commend the team to spend time in this important piece of evidence and further increase awareness of much needed multidisciplinary approach to a complex problem. The study is an investigation conducted in 2 regions in CAR with different endemicity and outbreak proneness related to mpox. Data was collected through interviews, participant observations and comparison with data from 30 years ago. Certainly, it is a long read and I wonder how much has been cut as well as not to bore the reader but keep them engaged. This is a type of study that requires the reader to step out of the strict qualitative research methodologies and appreciate the richness of information.

*We thank Reviewer 3 for this attentive and helpful evaluation of the manuscript. We recognize that it is a long read for a global public health journal, and indeed, we did cut a good deal of material for the first manuscript submission. That said, in response to other Reviewer questions, we have added some of this material (notably historical) back into the manuscript, though our great hope is that the manuscript retains reader attention.*

My suggestions to the research team to improve the manuscript are as follows:
- Line 25: the term mixed method -does this refer to quantitative and qualitative assessments or in just to say different methods were deployed?

*We had used the term mixed methods to refer to qualitative research conducted in 2022 and to the quantitative data collected in 1993 and analyzed in 2022-23. Nevertheless, because the bulk of our evidence is qualitative and because our use of the term mixed methods caused some confusion among reviewers, we decided to delete the term “mixed methods” altogether from the manuscript. We believe that this deletion does not change the manuscript in any substantial way and that it avoids confusion.*

- Line 30-31: a database of historical data is mentioned, but with one time point only i.e 30 years ago; is there no data from the time in between 1993 and 2022?

*No, unfortunately, no data (at least to which we have access) were collected between 1993 and 2022.*

- Line 33-34: The sentence ‘We also find important differences.’ belongs closer to Line 38 (.. reveals variability..) and not the similarity of the two sites in terms of their declension narrative

*Although we see what Reviewer 3 is suggesting, the first part of the Results in the Abstract refer to findings. The following material refers to the significance of our study. We have altered the language and inserted paragraphs to delineate better the different parts of our Abstract.*

- In the introduction, line 66 onwards – what is the ‘outbreak’ term refers here i.e what is the threshold of declaring it as outbreak in CAR? Is it a notifiable disease that must be reported to the health authority? Is one case enough to declare so?

*Our apologies for the lack of clarity. We have added “mpox” before outbreak to specify the type of outbreak (line 64). We have responded to the other questions on Lines 65-67, and in the Methods section in Lines 126-129.*

- Reference given in that paragraph is duplicate: reference 3 and 19 is the same

*Thank you for identifying this duplication. We have corrected it.*

- Line 102: The CAR – suggest removing the word ‘The’

*In our experience, the standard reference is “the CAR”, which is why we use it throughout the manuscript.*

#- Line 117-119: these 3 last sentences is unclear to read, suggest rephrasing

*We agree and thank the Reviewer for this point. We have modified these lines to clarify our point.*

On material and methods:

- Given the high mobility of the population, and the diversity of the tribes, how did selection bias be mitigated?

*We sought to contact a broad cross-section of people from different ethnic groups. In addition, we would note that the ethnic distinctions in these regions of CAR do not underpin substantive differences in livelihood practices. Different ethnic groups speak mutually intelligible or related languages, undertake similar livelihood practices, and share at least some underlying cultural practices and beliefs. Ethnic identity can be fluid and situation. For that reason, we would not even if we may not have reached every existing ethnic group in a region, we were able to interact with people from major ethnic groups and simultaneously to recognize (on the basis of long-term team expertise in this region) some shared characteristics across these groups.*

*Moreover, the overall point of this manuscript is to illustrate the differences across regions. Qualitative research does not seek to achieve representative samplings of certain populations, but rather to demonstrate a range of possible responses to a given set of queries. We are confident that our 40 qualitative interviews achieved that range.*

- What was the reason not to seek saturation during the data collection especially with sampling strategy of opportunistic participant recruitments and snowballing (asking key informants)

*The most important reason for not seeking saturation during data collection involved our limited time and budget for the field. That said, we were able to conduct 17 interviews in one site and 23 in a second, which is acceptable for a qualitative investigation.*

- How do you differentiate the reporting of individual versus group interviews or both are combined in Table 1? Why not doing focus group discussion?

*We opted not to do focus group discussions because given our limited time in the field, such discussions can be difficult to organize in light of agricultural and other livelihood demands on participants’ time.*

- Participant observation time seems quite short, would there be any reasons for this?

*Yes, we agree with Reviewer 3 on this point, and we would have liked to have spent more time doing participant-observation. Our time in the field was limited because of timing and budget constraints. We decided to privilege interviews over participant-observation. In addition, our research team included multiple, experienced Central African co-authors (Authors 2, 4, and 6), as well as Author 7, who has many decades of experience in CAR.*

- I appreciate clarity on the positionality and reflexivity of the research team

*Thank you*

- In results, the quotes are organized sometimes integrated in paragraph, sometimes not, please standardized (example: line 235, 258, 345..)

*We did not find guidance in PLOS GPH about the handling of quotes so we consulted recent publications for this guidance. We have now standardized the presentation of quotations throughout the manuscript.*

- Line 261 – is supplemental file 1 a text documenting rodent trapping techniques? (supplemental file I see is the video if not wrong)

*Supplemental File 1 is a video. We added some additional text in the Supplemental File to explain what the video depicts and why it is an important supplement to the manuscript.*

- Line 274 beyond – the description of eating meat habits may be more specific? Meat days means once per day? Per person or per family? Is it the size change of the animals hunted in 1993 the main thing we need to understand?

*We apologize for the confusion. We have clarified that a meat day is a day during which a participant in this survey consumed meat (Line 327). We explain more clearly the major result on lines 333-335.*

- Line 301 and elsewhere: is the word ‘moral’ used as ethical understanding that relates to religion or beliefs in this population?

*No, not exclusively. We use the term ‘moral’ in a more anthropological sense, referring to cultural standards or principles of what is socially good, appropriate, or bad. The term moral thus evokes an implicit code of conduct embraced by a society.*

- The discussion is well written but the implications can be theorized – e.g would paragraph starting line 419, would mean the messages at least to seek care should consider the nosological terms? Or would health workers be more aware and alert on the description of symptoms and also for notification of cases?

*We included a clearer explanation of our intent on lines 319-322. In addition, in response to this Reviewer’s evaluation, as well as those of other Reviewers, we have expanded on the broader implications of our work in the conclusion.*

- Please clarify if the Ebola messaging have influenced the way mpox is perceived or experienced being not lethal disease?

*We clarified this point in lines 519-522. But it is worth noting that mpox can be lethal, although not as lethal as Ebola.*

- The discussion is well written, albeit could benefit (missing text?) It is an interesting read and definitely help us appreciate the complexity of such disease ‘emergence’. Not the easiest read as well so I hope the feedback above can improve

*We sincerely thank Reviewer 3 for these comments, which have certainly helped us to clarify the manuscript. We have made every effort to ensure that it is more comprehensible to a wide range of readers.*

**REVIEWER #4:**

I want to thank the authors for putting together this insightful manuscript on “A time of decline: an eco-anthropological and ethnohistorical investigation of mpox in the Central African Republic”. Authors seek to explain why outbreaks may occur more frequently in some sites than others, or the processes and specific practices that may increase risks of transmission and accelerate the frequency of such outbreaks in recent years. This is done through anthropological and historical analyses of local knowledge of recurrent mpox outbreak and livelihood practices in CAR. However, needs major structural revision which will affect the scientific soundness.

**Abstract**: The abstract as its current state is difficult to comprehend. I suggest a structured format of writing an abstract be used. This will help readers better appreciate the content.

*We would also prefer a structured abstract. Nevertheless, we are following PLOS GPH guidelines, which indicate that structured abstracts should not be used.*

Introduction
Line 74, how has improved surveillance led to an increase and of what specifically?
Generally, epidemiological data on mpox outbreaks are missing. This data will be important to understand the ethnohistorical situation within CAR.

*We regret the unclear framing of this point and have therefore added the phrase “the increase in identified mpox cases” (Line 75).*

Materials and Methods

The structure is poor. No information on how data was managed, study period etc. Authors need to clearly document all steps using sub-headings.

*We agree with this criticism and have therefore added subheadings throughout this section to respond to this reader.*

Results
Can we have subheadings for all results presented?

*We do include subheadings throughout the results section. Please see lines 221, 263, and 353.*

Discussion.
This section is well written and backed by science but difficult to make judgement as to the soundness because other previous sections such as the method are not still vague.
Generally, the article will contribute greatly to global health security and prompt reproducibility for more findings that can help in the fight against zoonotic diseases.

*We thank Reviewer 5 for the careful read of this paper. In response to the criticisms of the Methods section, we have added additional structuring subheadings and have clarified certain aspects of the methods.*

*For clarity, we also added subheadings to structure better the Discussion.*

**REVIEWER #5:**

Broadly speaking, this article is an interesting read that fits with the aims and scope of the journal and nicely demonstrates the value of ethnographic research in a public health context. I look forward to being able to assign it to students in relevant courses.

My recommendations for revisions are relatively minor and mainly focus on clarifying the data, methods, and conclusions. First, I recommend including both a map indicating the study regions as well as Bangui and a table summarizing similarities and differences relevant to the analysis of the two study sites.

*We entirely agree and have added a map showing Bangui and the two study sites.*

Similarly, a figure of some kind in the discussion, such as a flowchart, showing broader conclusions of how different factors are related to each other and to health outcomes could be useful.

*We are deeply reticent about adding a flowchart here. Our anthropological and ethnohistorical data and analysis cannot be reduced to a set of factors and health outcomes. We collected insights from Central Africans about how they understand mpox and the reasons for the emergence of new diseases like mpox. Their knowledge, practices and insights fundamentally guided our analyses and are valuable in themselves. They cannot be distilled into factors contributing to transmission or a health outcomes. We have, however, added headings to organize the Discussion more clearly.*

Second, there are only minor and insufficient references to the supplemental file video. It is unclear what the purpose of including this video is and what readers should take from it, i.e., what it adds that the main text is unable to address.

*We have added additional explanation on Lines 304-305, as well as on Lines 547-549.*

Third, including details of the 1993 field research and related data with the rest of the data collection in the methods caused confusion for me. Beyond wondering how it was applicable to the current study and trying follow what data was from which collection period, I also then anticipated much more use of it in the later analysis. Personally, I think it would be fine to just briefly describe it the first time it is brought up in analysis/discussion, as one would with cited previous research, rather than highlight it with the main data collection.

*We recognize that the inclusion of this 1993 field data has caused some confusion. In the Methods, Results, and Discussion sections, we have modified how we refer to these data (cutting, for instance, the term mixed methods to reduce confusion). We respectfully contend, however, that it should be mentioned in the Methods so that readers know what data were collected and how. This explanation in the Methods section permits insight into the data and their limitations.*

Finally, I would like to see some additional discussion, if possible, of: a) differences in perspectives based on age, b) how the study could be used to improve public health communication and interventions. For example, what might a collaboratively developed preventive measure “look like”?

*We agree that age is important and have added limited text on lines 301-307 and 543-550, but in response to this and other reviewers’ comments, feel that we cannot make the manuscript any longer than it already is. Without adding too much text, Lines 823-826 have been added to suggest what such a collaboratively developed measure might be.*

On a minor point, there were a number of small grammatical errors, mostly missing or extra words, so be sure to review thoroughly. I do not include page numbers to indicate specific sections because the numbering was all messed up on my copy.

*Our apologies for both the unclear line numbering and for the small errors. We have reviewed the manuscript thoroughly for errors.*
