# Supplementary material for: A time of decline: An eco-anthropological and ethnohistorical investigation of mpox in the Central African Republic
Source: PLOS Glob Public Health. 2024 Mar 22;4(3):e0002937. doi: 10.1371/journal.pgph.0002937 (PMC10959331; doi:10.1371/journal.pgph.0002937)
Supplement: Attachment — Submitted filename: Peer review comments from Mateus_Mpox_R1.docx [file pgph.0002937.s005.docx]

**Peer review comments from M**

**Title:**

**A time of decline: an eco-anthropological and ethnohistorical investigation of mpox in the Central African Republic.**

**Abstract:**

OK

**Introduction:**

Line 69: … 99 confirmed … => I think this should be 99 confirmed cases.

**Materials and methods**

From Line 186-187: The following sentence is not clear: “Because of very high mobility among village residents in 1993, participants were contacted in person periodically over the study period (between June and December 1993)”. What is the study period (1993)? This is confusing because data were collected between 23 October to 30 November 2022.

Line 188-189: I don’t understand where this sentence is coming from: **“Data were therefore not collected for all 24 participants for the entirety of the six months data collection period”.** It does not have connection with the previous part. Why 24 participants? Where is this figure coming from?

**Results**

Line 268: Can authors give details about the socio-economic changes? What have changed socially or economically?

**Discussion**

OK

**Conclusion**

OK

**General comments:**

- Fix the issue of page number formatting
- The manuscript is scientifically relevant and carry the message that can inform policy and programs to improve public health interventions. Following the above review, the corrections are important to be made before further considerations.
