# Supplementary material for: A time of decline: An eco-anthropological and ethnohistorical investigation of mpox in the Central African Republic
Source: PLOS Glob Public Health. 2024 Mar 22;4(3):e0002937. doi: 10.1371/journal.pgph.0002937 (PMC10959331; doi:10.1371/journal.pgph.0002937)
Supplement: Attachment — Submitted filename: Response to Reviewer.docx [file pgph.0002937.s006.docx]

Below are our line-by-line responses to Reviewer 1’s comments along with a description of the specific modifications that we have undertaken in response to this reviewer’s remarks. Modifications described below are in blue and are underlined.

**Response to Reviewer 1**

Reviewer #1: Title:
A time of decline: an eco-anthropological and ethnohistorical investigation of mpox in the Central African Republic.

Abstract:
OK

Introduction:
Line 69: … 99 confirmed … => I think this should be 99 confirmed cases.

*Author response: To ensure clarity, we have added the word “cases” after “99 confirmed” on Line 69.*

Materials and methods
From Line 186-187: The following sentence is not clear: “Because of very high mobility among village residents in 1993, participants were contacted in person periodically over the study period (between June and December 1993)”. What is the study period (1993)? This is confusing because data were collected between 23 October to 30 November 2022.

*Author response: We regret the confusion. The anthropological study on which this manuscript is based did indeed take place between 23 October 2022 and 30 November 2022. This manuscript seeks to show changes over time in the type of available wild meat that could explain increased rodent consumption and thus exposure to potential animal reservoirs of mpox. Author 7 had conducted ethnohistorical and anthropological research in the same geographical region in 1993, and in this manuscript, we draw on two small surveys, previously unpublished, conducted during that period. Hence, this sentence refers to our use of the survey results in this manuscript. The survey data collected in 1993 is valuable because it permits us to compare changes in wild meat consumption between 1993 and 2022. Although the addition of this complementary evidence collected has caused some confusion, it is exceedingly rare to be able to mobilize this type of historical data.*

*To clarify our use of complementary data from this earlier investigation, we have therefore modified the text (see Lines 179-184):*

“In addition to conducting initial interviews in the Lobaye, Author 7 had previously conducted extensive ethnohistorical, anthropological field research in the Sangha-Mbaere in 1993. This article also analyzes data from a small complementary study conducted during this 1993 research, specifically from a local meat market survey and a daily activity, income, and 48-hour dietary recall survey to compare wild meat consumption with that during our 2022 study. The activity, income, and 48-hour dietary recall survey took place between June and December 1993 among 24 adults (14 men, 10 women) between approximate ages of 20 and 85 living in a major village (Lindjombo) in the Sangha Mbaere prefecture.

Line 188-189: I don’t understand where this sentence is coming from: “Data were therefore not collected for all 24 participants for the entirety of the six months data collection period”. It does not have connection with the previous part. Why 24 participants? Where is this figure coming from?

*This information about the 24 participants refers to the activity, income, and 48 hour dietary recall survey, conducted by Author 7 between June and December, 1993. It is part of a paragraph dedicated to this complementary survey that permit us to compare in this manuscript wild meat consumption between 1993 and 2022. Our modifications in Lines 179-184 clarify this point.*

*In response to Reviewer 1’s query “Why 24 participants?”, we offer two explanations:*

1. *We included 24 participants in the 1993 survey (out of a village of approximately 500 people) because Author 7 had very limited resources at the time and could only collect this data from this number of individuals. We indicate in the description of this survey that we used convenience sampling. Hence, Lines 186-189 explain as follows:*

Convenience sampling was used, and recruitment procedures sought to include at least one member from each major extended family living in the village. Individual participants were included for their willingness and availability to participate. Sample size was therefore not calculated.

2. *The overall manuscript is* ***qualitative****, so that the results of the survey are simply an indicator of past consumption, permitting us to compare how wild meat consumption has changed over nearly three decades. We make no claims to have obtained a statistically significant sample of people in a village, which we carefully note in the Methods section and Limitations of the study.*

*In response to Reviewer 1’s questions about the sentence “Data were therefore not collected for all 24 participants for the entirety of the six-month data collection period”, we apologize for the lack of clarity and have modified the sentence as follows.*

Data were therefore not collected each day for all 24 participants for the entirety of the six-month data collection period of this complementary study between June and December 1993.

Results
Line 268: Can authors give details about the socio-economic changes? What have changed socially or economically?

*The sentence beginning on Line 268 is simply an introductory sentence to the paragraph and to this section of Results. This sentence refers to “changing local socioeconomic, environmental, and seasonal* ***practices****”. Although we describe certain socioeconomic changes throughout this section, the focus in this section is on* ***the practices themselves*** *as a consequence of these changes. In response to the Reviewer’s remarks, we have modified in this first paragraph as follows:*

Our ethnohistorical, anthropological interviews also documented changing local socioeconomic, environmental, and seasonal practices that may have facilitated increased human-rodent physical contact and consumption. Alterations in hunting, trapping, and gathering practices, as well as changes in wild meat consumption have occurred in both study sites. These livelihood practices are important because they could bring inhabitants of the two sites into physical contact (through hunting, butchering, and meat preparation or handling for marketing) with an animal infected with the mpox virus.

Discussion
OK

Conclusion
OK

General comments:
- Fix the issue of page number formatting

*We have checked and rechecked the number formatting on the Word version submitted to the PLOS GPH site. The formatting is correct in the Word file that we have uploaded, but when the PLOS GPH site converts the submitted files into a PDF, the formatting is completely messed up. We have no control over this conversion, unfortunately, and have never had this problem before in submitting manuscripts to PLOS GPH or any other journal. We regret the inconvenience that this problem has caused for the Reviewer.*

- The manuscript is scientifically relevant and carry the message that can inform policy and programs to improve public health interventions. Following the above review, the corrections are important to be made before further considerations.

*Thank you for your time and care in reviewing this manuscript.*
